# Sensitivity and specificity of an eye movement tracking-based biomarker for concussion

**DOI:** 10.2217/cnc.15.3

**Published:** 2015-08-06

**Authors:** Uzma Samadani, Meng Li, Meng Qian, Eugene Laska, Robert Ritlop, Radek Kolecki, Marleen Reyes, Lindsey Altomare, Je Yeong Sone, Aylin Adem, Paul Huang, Douglas Kondziolka, Stephen Wall, Spiros Frangos, Charles Marmar

**Affiliations:** 1 Department of Neurosurgery, New York Harbor Health Care System, NY, USA; 2 Department of Neurosurgery, New York University, School of Medicine, NY, USA; 3 Steven & Alexandra Cohen Veterans Center for Post-Traumatic Stress & Traumatic Brain Injury, New York University Langone Medical Center, NY, USA; 4 Nathan Kline Institute for Psychiatric Research, Orangeburg, NJ, USA; 5 Oculogica Inc., NY, USA; 6 Department of Emergency Medicine, New York University School of Medicine, NY, USA; 7 Department of Trauma Surgery, New York University School of Medicine, NY, USA

**Keywords:** biomarker, concussion, eye movement tracking

## Abstract

**Object:**

The purpose of the current study is to determine the sensitivity and specificity of an eye tracking method as a classifier for identifying concussion.

**Methods:**

Brain injured and control subjects prospectively underwent both eye tracking and Sport Concussion Assessment Tool 3. The results of eye tracking biomarker based classifier models were then validated against a dataset of individuals not used in building a model. The area under the curve (AUC) of receiver operating characteristics was examined.

**Results:**

An optimal classifier based on best subset had an AUC of 0.878, and a cross-validated AUC of 0.852 in CT- subjects and an AUC of 0.831 in a validation dataset. The optimal misclassification rate in an external dataset (n = 254) was 13%.

**Conclusion:**

If one defines concussion based on history, examination, radiographic and Sport Concussion Assessment Tool 3 criteria, it is possible to generate an eye tracking based biomarker that enables detection of concussion with reasonably high sensitivity and specificity.

The complexity of diagnosis for concussion reflects many factors. First among these is the functional and anatomic variability of the normal brain. Even identical twins have differences in personality, behavior and abilities resulting in differential brain morphometry [1]. Children with poorer academic achievement scores may do worse on concussion detection tests that require reading [2]. For this reason, many concussion detection tests have required baseline assessments. A second factor is that the human brain is constantly changing over time. A classic example of this is the difference in functional MRI activated by speech as a child learns to read [3]. Tests requiring baselines are particularly vulnerable to developmental influence, learning curves, practice effect [4] and volitional exaggeration [5,6]. Third is the variability of brain injury itself. No two blows to the head can result in the exact same pattern of injury. A fourth factor is obfuscation by nonbrain injury and other factors which can result in headache, nausea, vomiting, dizziness and other symptoms mimicking brain injury.

Emergency department (ED) assessment of concussion patients generally includes history and physical examination, but can also include CT imaging, which does not quantitate concussion. Concussion may be a diagnosis of exclusion in the ED, or it may be overlooked, as the principal purpose of the ED visit is to ensure the absence of preventable morbidity and mortality.

The Sport Concussion Assessment Tool 3 (SCAT3) was designed to assess concussion signs and symptoms in athletes and has been validated in several studies, with subsets validated as SCAT2 [7–13]. Several other studies assessing diagnostics for concussion have relied on SCAT3 testing to assess the extent of the condition, and thus we have proceeded similarly [7–13]. We selected a symptom severity score (SSS) of >40 and standardized assessment of concussion score (SAC) ≤24 based on prior concussion studies with athletes and civilians [14,15]. This definition of concussion is consistent with Center for Disease Control descriptions of characteristics of concussion.

We have developed an eye tracking algorithm that detects cranial nerve palsies and is sensitive for detection of acute mass effect in the brain [16]. The algorithm also detects disruption of pathways controlling eye movements associated with structural traumatic brain injury (TBI) and concussion [17]. Eye tracking is performed while a subject watches television or a video moving inside an aperture with a set trajectory for 220 s at a fixed distance from a viewing monitor. The position of each pupil is recorded over time elapsed as the video travels on its time course, enabling detection of impaired ability to rotate the eyes relative to time and therefore relative to each other. In our previous work, we demonstrated that the severity of disconjugate gaze in ED structural TBI and concussion patients detectable with this algorithm was proportionate to the severity of concussion symptoms. Eye tracking also improved over time after both structural brain injury and concussion, with the former patients improving more slowly [17].

The purpose of the current study is to determine the sensitivity and specificity of our eye tracking metrics as a biomarker for concussion based on a classifier function.

## Methods

### Subject selection

Control subjects were employees, volunteers, visitors and patients at the Bellevue Hospital Center recruited in accordance with Institutional Review Board policy. Inclusion criteria for normal control subjects were: age 18–60 years, vision correctable to within 20/500 bilaterally, intact ocular motility and ability to provide a complete ophthalmologic, medical and neurologic history as well as medications/drugs/alcohol consumed within the 24 h prior to tracking. Exclusion criteria were history of: strabismus, diplopia, palsy of cranial nerves III, IV or VI, papilledema, optic neuritis or other known disorder affecting cranial nerve II, macular edema, retinal degeneration, dementia or cognitive impairment, hydrocephalus, sarcoidosis, myasthenia gravis, multiple sclerosis or other demyelinating disease, and active or acute epilepsy, stroke/hemorrhage or brain injury sufficiently significant to result in hospitalization. Subjects reporting any minor brain injury regardless of loss of consciousness were also excluded.

All trauma patients were recruited from the Bellevue Hospital Emergency Services (Emergency Room and Trauma Bay), trauma service and neurosurgery service. They were between the ages of 18 and 60, subject to the same exclusion requirements as controls except for head injury, consentable and able/willing to participate in the study. Both structural and nonstructural brain injury patients needed to have obtained a CT scan of the head prior to consideration for study enrollment. Trauma exclusion criteria included patients suffering burns, anoxic injury or multiple/extensive injuries resulting in any medical, surgical or hemodynamic instability. Structural brain injury was defined as final CT scan reading (by an attending physician radiologist) demonstrating the presence of hemorrhage (subdural, epidural, subarachnoid or intraparenchymal), brain contusion or full-thickness skull fracture consistent with acute brain injury. Structural brain injury patients were considered eligible for recruitment for up to 2 weeks after injury or surgery as long as they exhibited evidence of not yet being fully recovered from the brain injury (e.g., were still hospitalized.) No structural TBI patients were recruited preoperatively; they either had nonsurgical injuries or were recruited postsurgically. SCAT3 assessments were administered at the time of eye tracking by research personnel blinded to the eye tracking findings to patients blinded to their eye tracking results.

For the purposes of assessing eye movement as a biomarker for concussion, we defined concussion as: traumatic injury resulting in ED evaluation, sufficient indication for a CT scan of the brain, which was negative for structural brain or skull injury. Criteria for obtaining a head CT in the emergency room and trauma bay were based on Level One trauma center/ATLS/ACEP guidelines in accordance with the discretion of the individual examining physician responsible for the care of the patient. SCAT3 SSS of >40 and SCAT3 SAC score ≤24. Subjects meeting these four criteria were considered ‘true positives’ for concussion.

### Visual stimulus

We recorded subjects’ eye movements with an Eyelink 1000 eye tracker at a fixed distance of 55 cm from a computer monitor over a time period of 220 s. The distance was fixed by means of a chinrest attached to the base of the viewing monitor and camera. Subjects were seated in either a height adjustable or height-fixed chair or bed, with the monitor height adjusted to the subject as described previously [17]. The visual stimuli were the music videos Shakira Waka-Waka, K’naan Wavin’ Flag or Disney videos from Puss in Boots, Lion King or the Little Mermaid as per patient choice. The video was played continuously in a square aperture with an area approximately 1/8 the screen size while moving clockwise along the outer edges of a rectangular (aspect ratio 4:3) viewing monitor at a rate of 10 s per side for five complete cycles of 40 s each. The total visible span of the moving aperture was somewhat approximately 17º horizontally and 13º vertically from midposition with a caveat that the subject may be viewing different portions of the aperture during each cycle. The first and last 10 s of each dataset were discarded to yield 200 s of data. The afferent stimulus was presented binocularly and eye tracking was performed binocularly. Subjects were not spatially calibrated to the tracker to enable independent analysis of each pupil position over time.

### Data analysis

**Figure 1.**
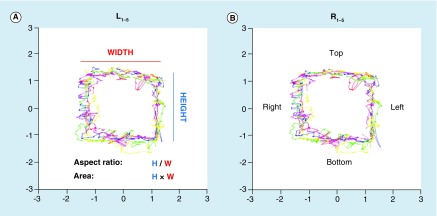
**Box plot trajectories of five cycles of eye movements for the left and right eyes over time.** Each cycle as the video plays continuously is shown in a separate color. L: Left; R: Right.

The eye tracker sampled pupil position at 500 Hz, yielding 100,000 samples over 200 s. We created scatterplots of the entire time series by plotting the 100,000 (x,y) pairs representing the two orthogonal components of the pupil position estimated by pupil–cornea reflection measurement over time to create ‘box trajectories’ that reflected the temporal nature of the pupillary movement. These figures look like boxes, reflecting the timing of the aperture as it moved around the screen (Figure 1) with each 10 s of data collection representing one unit of ocular traverse. Horizontally, the pupil traveled approximately 34° over 10 s and vertically it traveled approximately 23° in 10 s. Two-hundred data points prior to and following each blink were removed prior to creating the measures of disconjugacy and aspect ratio to limit noise in the data from the blink event.

Typical eye tracking experiments feature a gaze-point-fixation-based calibration system to train the eye tracker’s internal model to be able to accurately predict the subject’s gaze position on the screen. Our algorithm is not training a model eye gaze model nor is it concerned about the accurate localization of gaze on a screen. We do not need to account for error(s) of spatial gaze position as a subject views a particular point on the screen, but are rather interested in physiologic capability. Thus we take raw pupil coordinates from the EyeLink device transform the data based on values from each eye respectively and not mixing values across eyes, consistent with our assumption that brain injured patients have eyes that may not move together.

Without spatial calibration, exact measurements of error in the spatial domain are impossible. Our analysis avoided this problem by deeming it irrelevant what exactly the subject is viewing and assessing the eye movement trajectories in the time domain, rather than the spatial domain. By using a constantly changing stimulus (a continuously playing movie) with a periodic envelope (the aperture trajectory), we were able to look at relative eye movements over time. Effectively, each subject’s mean trajectory over the path of the aperture served as its own calibration. To clarify regarding the temporal nature of the boxes, consider an aperture that circles the perimeter of the monitor twice. At 38 s after the start of the time series, the eye tracker will report a pair of values, (x_1_,y_1_). Seventy-eight seconds after the start of the time series, the stimulus aperture will appear in the same spatial location as it did at second 38, and the eye tracker will report a second pair of values, (x_2_,y_2_). To be concrete, assume that the two pairs were (-2.0,0.1) and (-2.0,-0.1). The trial triggered average of the data (i.e., an average across the repeated cycles, synchronized by time of cycle start) would result in the pair (-2,0), and that is the point indicated in the box plot, even though no actual pupil angle measures corresponded to that pair of numbers. Further with each cycle it is not necessarily true that the pupils should be at the same exact Cartesian coordinate – on the contrary they are unlikely to overlap exactly given the size of the viewing aperture.

Eighty-nine separate metrics were obtained from these 100,000 data points obtained over time that reflected functions of individual and conjugate eye movements. Thirty-two of these metrics were assessing function of only the left eye, 32 of the right eye and 25 of both eyes. Metrics were named first according to whether they reflected function of a single eye (right or left) or both eyes (conjugacy) [16,17]. They were further subdivided as reflecting horizontal (x) or vertical (y) eye movement. Additionally they were then further subdivided by the location of the stimulus trajectory (right, bottom, left or top) or in all directions (total). For example some metrics were based on transformed pupil coordinates such as height or width of the box trajectory (Figure 1) which represented mean or median values of a pupil position over time, whereas others were calculated from these single metrics. Aspect ratio = height/width of the trajectory, which relates function of CNIII relative to CN VI [16]. Area = height x width and thus represents total function of CN III and VI. Distance was calculated using transformed (x,y) Cartesian coordinates and Pythagorean theorem. Velocity was distance over time. The BOX metrics were combinatorial scores calculated from raw metrics.

### Statistical analysis

True positives and negatives were age and gender balanced and their eye tracking metrics were compared using Bonferroni corrected Wilcoxon rank sum tests. To achieve a family-wise error rate of 0.05, a candidate biomarker must have a p-value below 0.05/89 = 0.00056. Ideally, a valid biomarker should be independent of gender or age. Accordingly, we evaluated the association between each eye tracking measure and age and gender in the full control sample and excluded those that were either significantly associated with gender (p ≤ 0.01) or age (p ≤ 0.05) for biomarker consideration. We built classifier functions using two model selection methods the ‘best subset’ model, and the least absolute shrinkage and selection operator method. To appraise the classifier, fourfold cross validation was repeated 1000-times to obtain an average AUC of the receiver operating characteristic (ROC) curve. We also utilized a random forests algorithm for obtaining a classifier. The random forests method builds a large bootstrap collection of logical trees, and then averages the individual predictions. An out-of-bag (OOB) error estimate is almost identical to that obtained by N-fold cross validation. The results of these eye tracking biomarker based classifier models were then validated against a dataset of individuals not used in building the model.

## Results

In order to generate the classifier models, we first considered both CT+ and CT- patients as a total group of brain injured subjects. The brain injured group had 42 subjects, 34 of which were males. Since there were only eight female cases, we decided to focus on male subjects. The current data had 281 control subjects, 129 of which were males. To balance age, we obtained a sample of 34 male controls and 34 male cases. In the selected sample, the age distribution of cases and controls are not significantly different (p = 0.801) (Table 1).

**Table 1.** **Summary statistics of age in the balanced sample.**

**Analysis variable : age**					

**Group**	**n**	**Mean**	**Standard deviation**	**Minimum**	**Maximum**
Control	34	41.55	12.61	21.12	62.15

Case	34	40.91	11.83	21	61

## Group comparisons

The 89 eye tracking measures of 34 controls and 34 brain injured cases were individually compared using the Wilcoxon rank sum test. The unadjusted p-values of selected measures are shown in Table 2. The eye tracking measures that remained significant after adjusting for multiple comparisons are shown in red below. It is likely that the number of significant variables would increase if we used more powerful multiple comparison adjustment methods, such as the bootstrap or Holm’s method.

**Table 2.** **Significant unadjusted p-values from among 89 eye tracking measures of contrasts of brain-injured and control subjects.**

**Variable**	**p-value**	**Variable**	**p-value**
left_areamean_value	0.023636	right_varXrit_value	0.043624

left_areamedian_value	0.010925	right_varYtop_value	0.030398

left_blinkrate_value	0.007223	right_widthmean_value	0.048997

left_distBot_value	0.000002	right_widthmedian_value	0.017049

left_distLef_value	0.000043	conj_boxscore_value	0.000057

left_distRit_value	0.000002	conj_boxscore2_value	0.001664

left_distTop_value	0.000027	conj_boxscore3_value	0.000029

left_nblinks_value	0.006833	conj_boxscore5_value	0.000075

left_varYbot_value	0.000334	conj_totVar_value	0.000907

left_varYtop_value	0.014904	conj_varAspect_value	0.001087

left_widthmean_value	0.026833	conj_varX_value	0.000217

left_widthmedian_value	0.004519	conj_varXbot_value	0.000217

right_areamedian_value	0.043624	conj_varXlef_value	0.001458

right_aspectRatiomedian_value	0.031350	conj_varXrit_value	0.000555

right_blinkrate_value	0.007223	conj_varXtop_value	0.000228

right_distBot_value	0.000384	conj_varY_value	0.005467

right_distLef_value	0.000002	conj_varYbot_value	0.014904

right_distRit_value	0.000089	conj_varYlef_value	0.010181

right_distTop_value	0.000001	conj_varYrit_value	0.017049

right_nblinks_value	0.006833	conj_varYtop_value	0.007096

## Biomarker generation

Among the 66 eye tracking measures that were not strongly associated with age or gender, 28 measures were found to be significantly different between controls and brain injured cases (p < 0.05). Four variables including conj_boxscore_value, conj_boxscore2_value, conj_boxscore3_value and conj_boxscore5_value were highly correlated, so only one, conj_boxscore_value was used for further model building. The p-values for comparing concussion cases versus controls, male versus female and correlating metrics to age and the area under the ROC curve (AUC) for each predictor are shown in Table 3.

**Table 3.** **The p-values and the area under the receiver operating characteristic curve (area under the curve) for each candidate eye tracking measure as it correlates to age, male versus female or concussion versus control.**

**Variable**	**p-value, correlated with age**	**p-value, male vs female**	**p-value, concussion vs control**	**AUC_value**
conj_boxscore_value	0.996	0.285	<0.0001	0.74

conj_totVar_value	0.122	0.25	0.0009	0.734

conj_varAspect_value	0.128	0.974	0.0011	0.733

conj_varX_value	0.856	0.074	0.0002	0.761

conj_varXbot_value	0.821	0.735	0.0002	0.761

conj_varXlef_value	0.948	0.223	0.0015	0.725

conj_varXrit_value	0.258	0.011	0.0006	0.744

conj_varXtop_value	0.93	0.095	0.0002	0.76

conj_varY_value	0.082	0.33	0.0055	0.696

conj_varYbot_value	0.624	0.69	0.0149	0.672

conj_varYlef_value	0.315	0.565	0.0102	0.682

conj_varYrit_value	0.057	0.256	0.0170	0.669

left_areamean_value	0.724	0.18	0.0236	0.66

left_areamedian_value	0.933	0.43	0.0109	0.68

left_varYbot_value	0.62	0.338	0.0003	0.753

left_varYtop_value	0.945	0.873	0.0149	0.672

left_widthmean_value	0.352	0.368	0.0268	0.657

left_widthmedian_value	0.439	0.79	0.0045	0.701

right_areamedian_value	0.118	0.523	0.0436	0.643

right_distTop_value	0.066	0.018	<.0001	0.848

right_varXrit_value	0.133	0.654	0.0436	0.643

right_varYtop_value	0.246	0.634	0.0304	0.653

right_widthmean_value	0.358	0.676	0.0490	0.639

right_widthmedian_value	0.102	0.834	0.0170	0.669

right_aspectRatiomean_value	0.854	0.453	0.0313	0.621

AUC: Area under the curve.

## Model building in the balanced sample (training data)

The age and gender balanced sample with 34 concussions and 34 controls was used to build the models. The selected variables, the AUC, cross-validated AUC, the misclassification rate and the cross-validated misclassification rate are shown in Table 4.

**Table 4.** **The model selection results in the balanced sample.**

**Method**	**Variables**	**AUC**	**Cross-validated AUC**	**Misclassification rate (%)**	**Cross-validated misclassification rate (%)**
Best subset	right_distTop_value, conj_varAspect_value, conj_varY_value, conj_varYlef_value	0.881	0.836	14.9	23.6

Best subset	right_distTop_value, conj_varX_value	0.870	0.856	17.6	23.9

LASSO	right_distTop_value, conj_boxscore_value	0.865	0.840	17.6	25.9

AUC: Area under the curve; LASSO: Least absolute shrinkage and selection operator.

We generated two models using the best subset approach due to a missing value of the conj_varAspect_value variable in a case subject. When we deleted that case with the missing value and performed the best subset approach, we obtained the model including four predictors. When we deleted the variable conj_varAspect_value and then performed the best subset approach, we generated the model with two predictors: right_distTop_value and conj_varX_value. The OOB misclassification rate for the random forest classifier is 27.9% using the 25 eye tracking measures shown in Table 3.

## Model validation in an external dataset

We tested the classifier performance in a validation dataset consisted of 255 subjects (247 mixed gender controls and eight female cases), which were not included in the all-male 34/34 training data. The misclassification rates, numbers of true positives, false positives, false negatives, true negatives, sensitivity, specificity and AUC of the three models are shown in Table 5. Note that random forest methodology does not enable a calculation of AUC.

**Table 5.** **The misclassification rates, numbers of true positives, false positives, false negatives, true negatives, sensitivity, specificity and area under the curve of the models.**

**Method**	**Variables**	**Misclassification rate (%)**	**TP (n)**	**FP (n)**	**FN (n)**	**TN (n)**	**Sensitivity**	**Specificity**	**AUC**
Best subset	right_distTop_value, conj_varAspect_value, conj_varY_value, conj_varYlef_value	22.4	7	56	1	191	0.875	0.773	0.827

Best subset	right_distTop_value, conj_varX_value	13.3	6	32	2	215	0.75	0.87	0.85

LASSO	right_distTop_value, conj_boxscore_value	18.0	6	45	2	202	0.75	0.818	0.841

Random forest	25 variables	23.1	6	55	2	192	0.75	0.777	–

AUC: Area under the curve; FN: False negative; FP: False positive; LASSO: Least absolute shrinkage and selection operator; TN: True negative; TP: True positive.

## Analysis on the balanced sample excluding CT+ subjects

In order to focus the biomarker on concussion, as opposed to including both concussion and structural brain injury, we removed the CT+ subjects from the balanced sample and redid the analysis. Age and gender are well balanced between the CT- cases and the controls in the balanced sample, while the validation dataset consisted of mixed-gender controls and female-only cases. The age distribution is not significantly different between CT- cases and controls (p-value = 0.665). Summary statistics of age in the CT- cases and the controls are shown in Table 6

**Table 6.** **Summary statistics of age in the balanced sample excluding CT+ subjects.**

**Analysis variable: age**					

**Group**	**n**	**Mean**	**Standard deviation**	**Minimum**	**Maximum**
Controls	34	41.55	12.61	21	62

CT- cases	21	43.33	10.49	24	61

We built three classifier models using the approaches described above for the balanced sample excluding CT+ subjects. The selected variables, the AUC, cross-validated AUC, the misclassification rate and the cross-validated misclassification rate are shown in the Table 7.

**Table 7.** **The model selection results in the balanced sample excluding CT+ subjects.**

**Method**	**Variables**	**AUC**	**Cross-validated AUC**	**Misclassification rate (%)**	**Cross-validated misclassification rate (%)**
Best subset	right_distTop_value, conj_varXbot_value	0.878	0.852	16.4	25.2

LASSO	right_distTop_value, conj_varXbot_value	0.880	0.826	16.4	26.9

AU: Area under the curve; LASSO: Least absolute shrinkage and selection operator.

The OOB misclassification rate is 23.6% using the 25 eye tracking measures shown in Table 3.

We tested the model performance in the validation dataset excluding CT+ subjects (247 controls and seven concussions). The misclassification rates, numbers of true positives, false positives, false negatives, true negatives, sensitivity, specificity and AUC of the models are shown in Table 8. Again note that random forest methodology does not enable a calculation of AUC.

**Table 8.** **The misclassification rates, numbers of true positives, false positives, false negatives, true negatives, sensitivity, specificity and area under the curve of the models in the validation data excluding CT+ subjects.**

**Method**	**Variables**	**Misclassification rate (%)**	**TP (n)**	**FP (n)**	**FN (n)**	**TN (n)**	**Sensitivity**	**Specificity**	**AUC**
Best subset	right_distTop_value, conj_varXbot_value	14.2	5	34	2	213	0.714	0.862	0.831

LASSO	right_distTop_value, conj_varXbot_value	13.8	5	33	2	214	0.714	0.866	0.833

Random forest	25 variables	13.0	4	30	3	217	0.571	0.879	

AUC: Area under the curve; FN: False neagtive; FP: False positive; LASSO: Least absolute shrinkage and selection operator; TN: True negative; TP: True positive.

**Figure 2.**
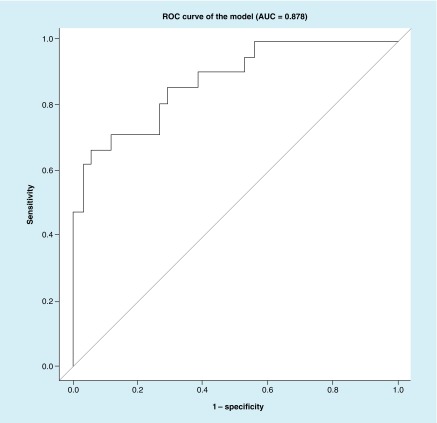
**An receiver operating characteristic curve of the best subset model.** Predictors in the model are: right_distTop_value and conj_varXbot_value. AUC: Area under the curve; ROC: Receiver operating characteristic.

We then created an ROC curve of the balanced sample excluding CT+ subjects (21 cases and 34 controls) using the best subset approach (Figure 2).

**Figure 3.**
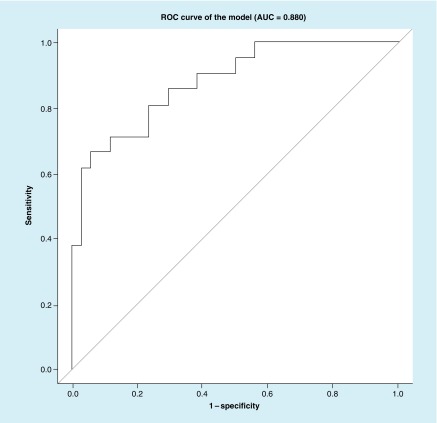
**Receiver operating characteristic curve of the least absolute shrinkage and selection operator model.** Predictors in the model are: right_distTop_value and conj_varXbot_value. AUC: Area under the curve; ROC: Receiver operating characteristic.

Then we created an ROC curve of the balanced sample excluding CT+ subjects (21 cases and 34 controls) using the LASSO approach (Figure 3)

**Figure 4.**
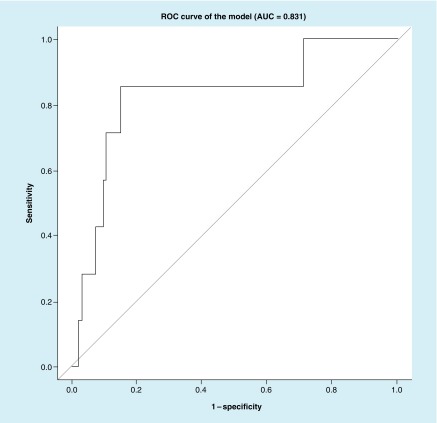
**Receiver operating characteristic curve of the validation sample using the best subset approach.** Predictors in the model are: right_distTop_value and conj_varXbot_value. AUC: Area under the curve; ROC: Receiver operating characteristic.

**Figure 5.**
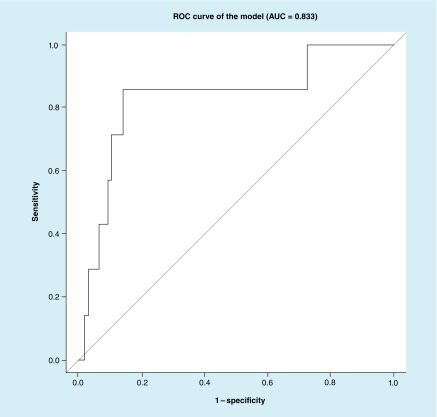
**Receiver operating characteristic curve of the validation sample using the least absolute shrinkage and selection operator approach.** Predictors in the model are: right_distTop_value and conj_varXbot_value. AUC: Area under the curve; ROC: Receiver operating characteristic.

We also created the ROC curves of the validation sample (247 controls and 7 concussions) using the best subset approach and LASSO approach (Figures 4 & 5).

## Discussion

Brain injury is known to have an impact on smooth pursuit, saccades, fixation, pupil size, vergence and other aspects of gaze [18–23]. Eye movement tracking for the assessment of brain injury has previously been performed in patients with postconcussive symptoms to assess both intrinsic ocular capability [24–26] and attention [27]. We have developed an algorithm that interprets eye tracking data obtained while a subject watches a music video, cartoon or other short film clip of their choosing as it moves in an aperture on a viewing monitor. The positions of the pupils are mapped over time and metrics are obtained assessing alterations in movement. The technology is rapid, noninvasive, automatable, portable and does not require literacy in any particular language. Its objectivity arises from the fact that it assesses relatively passive eye movements rather than requiring a subject to follow instructions and move their eyes deliberately.

Previously we have demonstrated that this algorithm detects both clinical and subclinical cranial nerve palsies resulting from both direct nerve damage, and from intracranial mass effect in the supra- and infra-tentorial spaces. The ocular motility deficits were found to be reversible with correction of the neurosurgical problem [16]. We have also shown that brain injured subjects have greater ocular dysmotility than nonbrain injured subjects, while nonhead-injured trauma subjects are not different from nonbrain-injured subjects. The severity of ocular motility dysfunction correlates with the severity of concussion symptoms in trauma subjects regardless of whether that injury can be seen on CT imaging. Deficits are worse in the 1–2 weeks after the injury and then recover in most patients at about 1 month postinjury [17].

Criticism [28] of our prior work with eye tracking of brain injured subjects focused on four points, which we address individually:

- “Any asymmetry in the spatial relationship that the camera or the infrared light source has with the two fellow eyes would result in different extents of relocation of the images of the pupils or corneal reflections. Asymmetries exist because there is a physical separation between the two eyes as well as between the camera and the infrared light source.” Asymmetry in the spatial relationship between camera, light source and eyes was controlled by using a chin and forehead rest fixed to the base of the viewing monitor and camera. By fully constraining this system, asymmetries were reduced. While there is physical separation between the eyes, this distance is a constant value in any given individual. As C Tyler explained [28] in his refutation of the Maruta comments, if these asymmetries were an issue, “all patients would be equally subject to the same degree by the effects of asymmetry and lack of calibration. As stated, none of the criticisms suggest a systematic bias between the different patient categories. The significant differences among categories cannot therefore be attributed to any of the factors raised by the author, and controlling these factors should only improve the significance of [the] result”;
- “[…although] eyes are highly symmetrical within individuals, they are not perfectly symmetrical^2^ and a 1–2% nonconformity in corneal curvature or axial length is not uncommon, which further confounds the relationship between eye rotation and changes in pixel coordinates… mapping is not linear.” Again the above refutation applies – such a problem should affect controls as much as trauma subjects. In addition, our current paper describes numerous significant metrics not relying on measurements from both eyes, but rather from a single eye;
- “Implementing a calibration procedure under monocular viewing” would achieve the same purpose as our algorithm. While this criticism is technically correct, it has been our experience that trauma patients are willing to watch a film clip for 220 s, but somewhat less willing to sit through an additional 5 min of monocular calibration despite the fact that this can easily be performed with a monocular occluder;
-  “Having a larger male-to-female ratio in one group could increase the extent of binocular asymmetry in uncalibrated data since men tend to have a larger interpupillary distance.” In this current work, we present data demonstrating that there is no difference in horizontal conjugacy between male and female subjects.

## Conclusion

In the current study, we establish that numerous parameters vary between brain injured subjects and controls (Table 2), and that some of these parameters are relatively independent of age and gender (Table 3). Ultimately we establish a relatively high sensitivity and specificity of this eye tracking algorithm for classifying concussion (Figures 1 & 2; Tables 7 & 8). Interestingly concussion had higher misclassification in the balanced sample (Table 7) than in the larger external validation dataset (Table 8). We suspect this may be because the balanced sample had patients who obligatorily had particular SCAT3 subset scores, which may imperfectly correlate with actual brain functionality. This misclassification rate also reflects a limitation of our methodology: specifically that there is currently no ‘gold standard’ diagnostic for concussion. Thus, generation of an AUC relies on our defining ‘true positives’ for concussion using best available standards. The SCAT3 SSS and SAC are at present the most widely validated measures for concussion. Data suggesting that some patients may maintain cognitive functioning even in the presence of structural brain injury underscores the complexity of brain function and injury [29]. A different patient with the same injury may have higher or lower functional cognitive assessment dependent on baseline capabilities. Thus, some of the ‘misclassification’ associated with our classifier may be due to the inadequacy of the SCAT3 subcomponents rather than of eye tracking.

Additional limitations of our study are that the validation data set of 7 concussions in 254 subjects is relatively small and that the control group excluded individuals with prior recent brain injury. If the eye tracking metrics are highly interdependent, the chance of type II errors becomes higher with corrections for multiple comparison. Also trauma patients had to have obtained a head CT to participate in the study, which may potentially imply that they were more severely injured than many concussion patients who do not receive a head CT.

Finally, we have the limitation of having a relatively large misclassification rate but this limitation is rendered less clinically dangerous due to the fact that most misclassifications are false positives. With our algorithm, to identify one true positive, six to eight negative people are classified falsely as positive. Since the medical risk of missing a case is greater than the risk of falsely classifying a negative patient as concussed this may be an acceptable risk. Consider for example, the imbalanced number of concussions and controls in our external validation dataset. One could imagine that a hypothetical model which randomly classifies everyone as normal would only have a 3% misclassification rate in an imbalanced sample such as ours. However, such a model would miss all seven concussions and thus hardly be optimal for patients.

The complexity of concussion does not lend itself well to a single diagnostic. Our eye tracking algorithm appears to be detecting at least two parameters: intracranial mass effect and disruption of neural pathways controlling ocular motility. It is logical to assume that ‘concussions’ not affecting these parameters will not be detectable with our algorithm.

While our current results are promising, additional data on potential confounders of eye tracking still need to be investigated. These include alcohol and other intoxicants, fatigue and prior history of trauma and neurologic or ophthalmic disorders among others. Future studies currently in progress will elaborate the role of these factors on eye tracking as a biomarker for concussion.

**Executive summary**

- Concussion is a condition that is not well defined; therefore it is difficult to diagnose.
- The purpose of this work is to determine the sensitivity and specificity of an eye movement tracking based biomarker for concussion.

**Methods**

- Brain injured subjects recruited through the Bellevue Hospital emergency department and normal uninjured controls were prospectively enrolled in a study in which both eye tracking while watching a short film clip for 220 s and Sport Concussion Assessment Tool (SCAT3) data were collected.
- For the purposes of assessing eye movement as a biomarker for concussion, we defined concussion as traumatic injury resulting in emergency department evaluation, sufficient indication for a CT scan of the head, which was negative for structural brain or skull injury, SCAT3 symptom severity score of >40 and standardized assessment of concussion subset of SCAT3 ≤24.
- True positives and negatives were age and gender balanced and their eye tracking metrics were compared using Bonferroni corrected Wilcoxon rank sum tests.
- We built classifier functions using two model selection methods the ‘best subset’ model, and the least absolute shrinkage and selection operator (LASSO) method. We also utilized a random forests method of obtaining a classifier.
- The results of these eye tracking biomarker based classifier models were then validated against a dataset of individuals not used in building the model.

**Results**

- Significant group differences between brain injured and concussed subjects versus negative controls were found for 28 eye tracking metrics that were not influenced by age or gender. These were used to develop the three classifier functions.
- In a sample of 21 concussion cases versus age and gender balanced uninjured controls, the ‘best subset’ model selected four metrics and the resulting receiver operating characteristic of the classifier had an area under the curve (AUC) of 0.878, and a cross-validated AUC of 0.852. The LASSO model selected two metrics and resulted in an AUC of 0.880 and a cross-validated AUC of 0.826.
- In an external dataset of 254 subjects (247 controls and sevenconcussions), ‘best subset’ had a misclassification rate of 14.2%, LASSO had a misclassification rate of 13.8% and random forest had a misclassification rate of 13.0%.

**Discussion**

- If one defines concussion based on history, physical examination, radiographic and SCAT3 criteria, it is possible to generate an eye tracking based biomarker that enables detection of concussion with reasonably high sensitivity and specificity.
